# On the advantages of using AI-generated images of filler faces for creating fair lineups

**DOI:** 10.1038/s41598-024-63004-z

**Published:** 2024-05-29

**Authors:** Raoul Bell, Nicola Marie Menne, Carolin Mayer, Axel Buchner

**Affiliations:** Department of Experimental Psychology, Heinrich Heine University Düsseldorf, Düsseldorf, Germany

**Keywords:** Generative artificial intelligence, AI image generation, Lineup fairness, Simultaneous lineups, Sequential lineups, Psychology, Human behaviour

## Abstract

Recent advances in artificial intelligence (AI) enable the generation of realistic facial images that can be used in police lineups. The use of AI image generation offers pragmatic advantages in that it allows practitioners to generate filler images directly from the description of the culprit using text-to-image generation, avoids the violation of identity rights of natural persons who are not suspects and eliminates the constraints of being bound to a database with a limited set of photographs. However, the risk exists that using AI-generated filler images provokes more biased selection of the suspect if eyewitnesses are able to distinguish AI-generated filler images from the photograph of the suspect’s face. Using a model-based analysis, we compared biased suspect selection directly between lineups with AI-generated filler images and lineups with database-derived filler photographs. The results show that the lineups with AI-generated filler images were perfectly fair and, in fact, led to less biased suspect selection than the lineups with database-derived filler photographs used in previous experiments. These results are encouraging with regard to the potential of AI image generation for constructing fair lineups which should inspire more systematic research on the feasibility of adopting AI technology in forensic settings.

Artificial intelligence (AI) has dramatically improved, leading not only to the capacity to generate human-like language^1^ but also to a significant enhancement in the quality of AI-generated images^2,3^. The capacity of AI to generate realistic images is now accessible through freely available and commercially licensed software. Much like the introduction of any novel technology^4,5^, the dramatic improvement in AI image generation has sparked widespread concerns. Specifically, concerns have been raised that, with the help of AI image generation, it is possible to effortlessly produce highly realistic images of persons that are difficult to distinguish from real photographs, which could be misused for launching harmful disinformation campaigns^2,6^. While the concerns about the challenges and risks arising from the advances of AI-generated output may seem almost limitless, the same holds true for potentially beneficial applications. Here we focus on a specific application of AI-generated images: the generation of facial filler images for police lineups.

A police lineup is conducted to either affirm or challenge the hypothesis of a suspect’s involvement in a crime^7^. The lineup essentially functions as a memory test designed to establish whether an eyewitness can recognize the culprit. Lineups are often conducted using photo arrays due to their cost effectiveness and feasibility compared to live lineups. Detailed guidelines exist on how to conduct these lineups^8^. Importantly, a photo lineup does not only comprise the suspect’s photograph but also photographs of several fillers who are unequivocally known to be innocent. The advantage of using fillers is obvious: When eyewitnesses guess rather than rely on memory, they do not inevitably select the suspect who, if innocent, would then be mistaken for the culprit, but may select a filler^9^. Since the fillers are known to be innocent, falsely selecting a filler has less severe consequences than falsely selecting an innocent suspect. To minimize the likelihood of an innocent suspect being falsely selected, the lineup has to be fair^10^. To achieve a high level of lineup fairness, the characteristics of the filler photographs are crucial. Ideally, the fillers should qualify as potential culprits just like the suspect does. If the lineup is perfectly fair, the suspect should be distinguishable from the fillers only based on the eyewitness’ memory for the face of the culprit.

Creating a fair lineup is more difficult than it might seem at first glance^11^. First, it must be ensured that the fillers match the culprit’s description to the same degree that the suspect does. Second, the suspect’s photograph must not differ in any other characteristics—such as color, contrast or brightness—from the filler photographs so that the suspect cannot be identified without relying on memory for the culprit. These points may sound trivial but are in fact difficult to consistently implement in practice. The photograph of the suspect often stems from a different source than the photographs of the fillers: While the photograph of the suspect could be a mugshot or (if the suspect is not already in police custody) a social-media picture, the photographs of the fillers are taken from special databases. In addition, sometimes the suspect has a distinctive facial feature (for example, a scar or a tattoo) that makes it very hard, if not impossible, to find fillers who share that specific feature. In these cases the photographs of the fillers must be digitally modified to eliminate such differences between the suspect photograph and the filler photographs^12^. These challenges are not only faced by police practitioners but also by researchers aiming to create fair lineups for research purposes^13,14^.

Here the recent advances in AI image generation may prove useful. Specifically, AI-generated filler images may offer some pragmatic advantages for the construction of lineups.

1. The images of fillers can be generated directly from the description of the culprit: Guidelines sometimes include the recommendation that fillers should be selected based on their resemblance to the description of the culprit provided by the eyewitness rather than on their resemblance to the suspect’s appearance^8^. The reason for this recommendation is that selecting filler faces that closely resemble the suspect can negatively affect the detection of the culprit in the lineup^15,16^. If, in an extreme example, all faces in the lineup look almost identical, there are hardly any unique facial features that can be used to recognize the culprit. By contrast, if the fillers are selected based on their resemblance to the description of the culprit, the fairness of the lineup is preserved (because any member of the lineup could potentially be the culprit) but the features that vary among the faces can be used to detect the culprit. Since AI technology opens up the possibility of text-to-image generation, the filler faces can be generated directly from the description of the culprit.
2. Identity rights are protected: To avoid violating identity rights, some countries, including some federal states of Germany, have rules against using the faces of natural persons as fillers^17^. To comply with these legal requirements, real faces are digitally altered, for example, by morphing different faces together so that the resulting filler faces no longer correspond to the faces of natural persons^14^. AI image generation offers a straightforward way to generate filler faces that are based on the descriptions of real faces but cannot be assigned to specific natural persons.
3. An unlimited number of faces can be generated: If the filler faces are selected from a database, then it is inevitable that this database will be limited to a certain number of photographs. AI image generation does not have this limitation because the number of filler faces that can be generated is limited only by the available computing power.

Even though AI-generated images have already reached an impressive level of realism, the suitability of AI-generated images for constructing fair lineups remains uncertain due to subtle yet potentially noticeable deviations from real faces, such as variations in skin and hair texture, hyper-realistic exaggerations of details and atypical facial asymmetries. If eyewitnesses notice these peculiarities, the real photograph of the suspect may stand out among the AI-generated filler images, thereby provoking a biased selection of the suspect. To test whether this is the case, we used a multinomial processing tree model^18^. Multinomial processing tree models are prevalent in memory research to separate memory and guessing processes^19–23^. In the present application, the two-high threshold (2-HT) eyewitness identification model^13,24^ was used to measure the biased selection of the suspect directly from eyewitness decisions. This measurement model serves to estimate the probabilities of four processes, two memory-based processes—culprit-presence detection, culprit-absence detection—and two non-memory-based processes—guessing-based selection and, most importantly here, biased suspect selection. To achieve this, the model utilizes all of the response categories that are observed in lineups: correct culprit identifications, false identifications of innocent suspects, false and correct rejections as well as filler identifications in culprit-present and culprit-absent lineups. The model is illustrated in Fig. 1.

**Figure 1:**
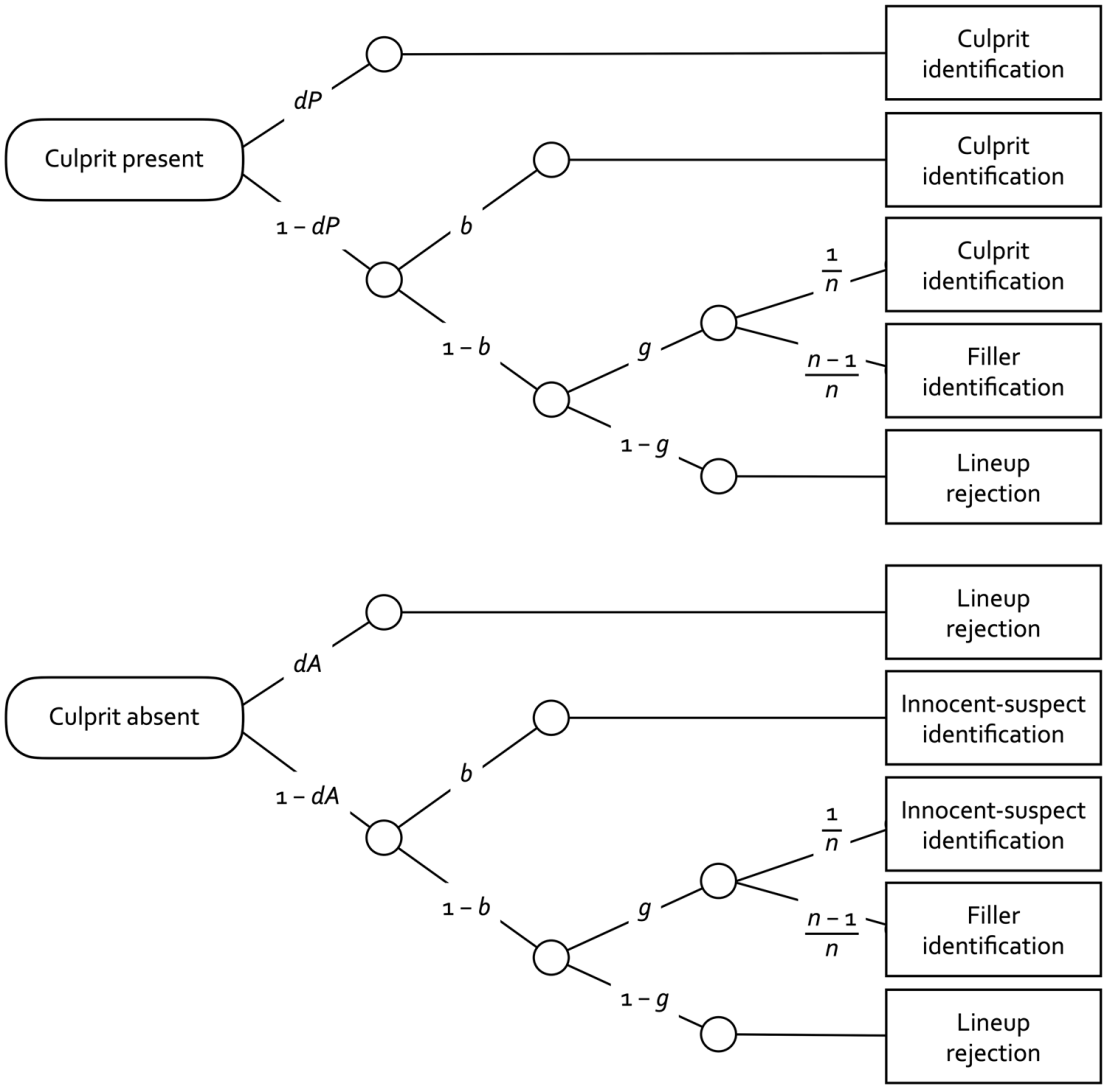
The 2-HT eyewitness identification model illustrated in the form of processing trees. The rounded rectangles on the left side represent the stimuli that are presented: culprit-present lineups (upper tree) and culprit-absent lineups (lower tree). The rectangles on the right side represent the different categories of responses that can be observed in these lineups. The letters along the branches represent the probabilities of the postulated latent processes (*dP*: culprit-presence detection; *b*: biased suspect selection; *g*: guessing-based selection among the lineup members; *dA*: culprit-absence detection). The sampling probability of randomly selecting the suspect among the lineup members in case of guessing is given by the reciprocal of *n*, where *n* refers to the lineup size.

The upper tree depicted in Fig. 1 illustrates the processes that are postulated to occur when eyewitnesses are presented with culprit-present lineups. The presence of the culprit is detected with probability *dP*, resulting in a correct culprit identification. This process has been shown to be sensitive to manipulations that affect the encoding and retrieval of the culprit’s face^13,24^. If the presence of the culprit is not detected which occurs with the complementary probability 1 − *dP*, the culprit can still be selected based on two non-detection based processes. Most importantly in the present context, biased selection of the culprit may occur with probability *b* if the photograph of the culprit conspicuously differs from the filler photographs. For example, parameter *b* is enhanced if the culprit has a distinctive facial feature such as a tattoo that makes the culprit’s face stand out from the filler faces^13,14,24^. With the complementary probability 1 − *b,* no biased selection occurs. In this case eyewitnesses may still select one of the lineup members based on guessing with probability *g*. For example, lineup instructions that imply that the culprit is in the lineup lead to increased guessing-based selection^13,24^. Guessing-based selection leads to a random selection among the lineup members. The sampling probability of selecting the suspect in a six-person lineup is one out of six because there is only one culprit that can be sampled. The complementary probability of selecting one of the fillers is five out of six because there are five fillers that can be sampled. With the complementary probability 1 − *g*, eyewitnesses may not make a guessing-based selection, which leads them to falsely reject the lineup. The lower tree depicted in Fig. 1 illustrates the processes in response to lineups in which the culprit is absent and instead an innocent suspect is included. The absence of a culprit is detected with probability *dA*, resulting in a correct lineup rejection. This process is facilitated, for instance, if all lineup members in the culprit-absent lineups (including the innocent suspect) can easily be ruled out because of a distinctive facial feature that distinguishes them from the culprit^13^. If the absence of the culprit is not detected, which occurs with the complementary probability 1 − *dA*, biased selection and guessing processes occur with the same probabilities as in the culprit-present lineups but here they refer to the innocent suspect in lieu of the culprit. For example, if an innocent suspect has a distinctive facial feature that distinguishes the innocent suspect’s face from the filler faces, then this may lead to the biased selection of the innocent suspect in a culprit-absent lineup with probability *b* just as this may lead to the biased selection of the culprit in a culprit-present lineup. Likewise, if an eyewitness chooses one of the lineup members based on guessing with probability *g*, then this may lead to the random sampling of the innocent suspect in culprit-absent lineups just as this may lead to the random sampling of the culprit in culprit-present lineups.

For the research question examined here, it is a crucial advantage of the model^13,24^ that it has been demonstrated through experimental validation and reanalyses of previously published data that the biased-suspect-selection parameter *b* can sensitively capture the unfairness of lineups. In the experimental validation^13^, unfair lineups were created in which all faces except that of the suspect had large facial birthmarks. In the fair control condition, none of the faces had large facial birthmarks. The biased-suspect-selection parameter was increased in the unfair-lineup condition compared to the fair-lineup condition. Analogously, reanalyses of previously published data^24^ have shown that biased selection of the suspect is increased compared to a fair control condition when the lineups are unfair because poor fillers are used that are dissimilar to the culprit^25^. Moreover, biased suspect selection is increased when the suspect stands out because only the suspect’s face has a distinctive facial feature (scar, bruising, piercing and tattoo) compared to the fair control condition in which the distinctive facial feature of the suspect is masked or replicated in the filler faces^12^. Another series of experiments has shown that subtle morphing artifacts also lead to increased biased suspect selection, but only when the instructions explicitly prompt participants to select the photographs that conspicuously stand out in the lineups, which is in direct opposition to established guidelines for real-world lineups^14^. The effects of lineup fairness are not always limited to the biased selection of the suspect: When participants focus on a suspect that conspicuously stands out from the lineup, guessing among the lineup members is decreased because the poor quality of the fillers discourages the random selection among the lineup members^13,14,24^.

The main purpose of the present experiment was to assess biased suspect selection in lineups with AI-generated filler images. As a benchmark, we used the lineups with database-derived filler photographs that were utilized in fair-lineup conditions in previous experiments^13,14^. The direct comparison of lineups with AI-generated filler images to those already comparatively fair lineups with database-derived filler photographs represents a deliberately strict criterion for judging the usefulness of the AI-generated filler images for creating fair lineups. If the AI-generated filler images differ to a greater degree from the suspect than the database-derived filler photographs, biased selection of the suspect should be increased in the lineups with AI-generated filler images compared to the lineups with database-derived filler photographs. By contrast, if the AI-generated filler images are useful for creating fair lineups, then biased suspect selection in the lineups with AI-generated filler images should be at the same level as, or even lower than, biased suspect selection in the lineups with database-derived filler photographs.

## Methods

### Participants

The experiment was conducted using the online tool SoSci Survey^26^. Participants were recruited using the research panel of Cint (https://cint.com). We aimed at a sample size of at least 750 valid data sets and ended data collection at the end of the day this criterion was reached. Of the data of the 931 participants who completed the socio-demographic questionnaire at the start of the experiment, 136 had to be excluded from the analysis because the participants had not completed the experiment or had withdrawn their consent to the use of their data. Data of 11 participants had to be excluded because these participants had accessed the experiment and had seen the video with the culprits twice. Data of 18 participants had to be excluded because these participants had failed the attention check (see *Procedure* section). The final data set contained data of *N* = 766 participants (319 female, 446 male, 1 non-binary) with a mean age of 45 (*SD* = 15) years. The sample was characterized by a diversified level of education. A sensitivity analysis with G*Power^27^ showed that, with a sample size of *N* = 766 and four eyewitness decisions per participant, an effect of filler type (AI-generated images, database-derived photographs) on biased suspect selection (*df* = 2) as small as *w* = 0.07 could be detected at an α level of 0.05 with a statistical power of 1 − β = 0.95. Participants were randomly assigned to the four conditions: simultaneous lineups with AI-generated filler images (201 participants), simultaneous lineups with database-derived filler photographs (184 participants), sequential lineups with AI-generated filler images (184 participants) or sequential lineups with database-derived filler photographs (197 participants).

### Ethics statement

Informed consent was obtained from all participants. Approval was received from the ethics committee of the Faculty of Mathematics and Natural Sciences at Heinrich Heine University Düsseldorf for a series of experiments including the present experiment. Participants were warned that they would see a short video that included verbal and physical abuse and were asked not to proceed if they felt uncomfortable expecting to watch such a video. At the end of the experiment, the participants were thanked for their participation and the purpose of the experiment as well as the fact that the crime depicted in the video had been staged was disclosed. All methods were performed in accordance with the relevant guidelines and regulations.

### Materials

We used the same mock-crime videos, the same facial photographs of the suspects and the same database-derived facial filler photographs as in our previous experiments^13,14,28,29^. The facial images of the AI-generated fillers were newly created for the purpose of the present experiment. The suspect and filler photographs showed the faces from a frontal view with a neutral facial expression. In our previous studies using database-derived filler photographs^13,14,28,29^, the suspects’ faces and the filler faces were cropped from the original photographs, were placed on a black background and their color and contrast were individually adjusted to equate color, contrast and backgrounds between the suspects’ photographs and the database-derived filler photographs. Furthermore, all photographs had been shown with a resolution of 142 × 214 pixels to facilitate the online presentation of the faces. The same cropping, background standardization, color and contrast adjustments and resolution specifications were applied to the AI-generated filler images in the present study to keep these aspects of the procedure constant when comparing the lineups with database-derived filler photographs to the lineups with AI-generated filler images.

#### Mock-crime videos and suspect photographs

As in our previous experiments^13,14,28,29^, there were two mock-crime videos that were parallel to each other. In each of the two videos, four different culprits were shown. Showing multiple culprits served the primary aim of improving the sensitivity of the statistical tests by increasing the number of observations obtained per participant. However, the scenario is also representative of real-world crimes that frequently involve multiple culprits^30–32^. In both videos a victim, dressed in fan clothing of the German soccer team Borussia Dortmund, was harassed and beaten by four culprits, dressed in fan clothing of the German soccer team FC Bayern München [for a more detailed description of the incident, see^13^]. In each of the two parallel videos, the sequence of events followed the same chronology but the culprits and the victims were played by different actors. Each culprit in Video A matched one of the culprits in Video B in basic features such as hair color, hair style and body type. The videos had a resolution of 885 × 500 pixels, lasted 130 s and offered an extended view of the culprits’ faces from different angles. Color portrait photographs were taken from each of the culprits of each video.

#### Database-derived filler photographs

The database-derived filler photographs were the same photographs that had been used in our previous experiments^13,14,29^. The photographs were taken from the Center for Vital Longevity face database^33^. The fillers in each lineup, white men between 18 and 29 years, were selected to match the suspect in physical features such as hair color, hair style and body type.

#### AI-generated filler images

Adobe Photoshop 24.7 (beta) was used to generate the AI-generated filler images. The images were created with four different text-based prompts generated by the present authors, each containing a description of one of the four culprits. For example, one of the culprits was described as “a young white male, without beard, medium-length dark-brown hair, slim”. The description thus incorporated the same aspects that determined the selection of the database-derived filler photographs in our previous studies^13,14,28,29^. We also included in the prompt that the face should have a neutral facial expression, should be displayed in the center of the image and was photographed indoors. Technical details of a virtual camera were also included to improve the quality of the AI-generated facial images. If an image contained obvious artifacts, we used the advantage of not being limited to a specific number of images by discarding and generating new images until we achieved a satisfying quality. The facial images of all four lineups are available at the OSF project page (see data availability statement).

### Procedure

We used the same procedure as in our previous experiments [for more detailed descriptions, see^13,14,29^]. Participants saw one of the two parallel videos described above. After participants had seen the complete video, they were asked to answer an attention-check question. Participants passed the attention check if they selected, from a randomized list of ten alternatives, the answer that the video had been about “soccer fans”.

Next, participants received the pre-lineup instructions. They were asked to identify the culprits. They were informed that several lineups would follow, each consisting of different faces. Two-sided instructions were used in which participants were asked to identify the culprit but also to reject the lineup if the culprit was absent [cf.^34^].

Participants were then shown four separate lineups, presented in a random order. Depending on the condition, the lineups consisted of a photograph of the suspect’s face and of either five AI-generated facial filler images or five database-derived facial filler photographs. The lineups were preceded by labels indicating the lineups’ ordinal numbers (“1st lineup”, “2nd lineup”, …). Two of the four lineups were randomly selected to contain a culprit. The remaining two lineups contained an innocent suspect. The innocent suspect was one of the actors from the video participants had not seen. For instance, if participants had seen Video A, then two randomly selected actors from Video A (for example, Actors 1 and 3) served as culprits while two actors from Video B (in this example, Actors 2 and 4) served as innocent suspects. Given that it had been randomly determined which of the two parallel videos had been seen, each of the actors was equally likely to be presented as the culprit or innocent suspect. This crossed-lineup procedure ensured that, on average, the culprits and innocent suspects differed to the same degree from the fillers^13,14,29^. The positions of the faces in the lineup were randomly determined.

#### Simultaneous lineups

In the simultaneous lineups condition, the participants were shown the faces of the suspect and the fillers together in a single row. Underneath each face, a button labeled “Yes, was present” could be selected to identify a face. Alternatively, participants could select a “No, none of these persons was present” button to the right of the lineup. Participants could take as much time as they required to arrive at a response. Participants had to confirm their response by clicking on a “Continue” button at the right side of the screen. After a click on this button, the next lineup was shown or, if participants had already seen all four lineups, they were transferred to a final webpage. On this page, participants were thanked for participating and the purpose of the experiment as well as the fact that the crime in the video had been staged were explained to them.

#### Sequential lineups

In the sequential lineups condition, the faces were presented one at a time. Participants had to decide for each face whether or not it belonged to one of the culprits by selecting either a “Yes, was present” button below the face or a “No, this person was not present” button presented to its right. Participants could take as much time as they required to arrive at a response. Participants had to confirm their response by clicking on a “Continue” button at the right side of the screen. After a click on this button, the next face was shown. When all six faces of a lineup had been presented, the next lineup started unless participants had already seen the fourth lineup in which case they were transferred to the same final webpage as participants in the simultaneous lineups condition. In many lab experiments with sequential lineups, participants were informed that only their “first yes counts” and the presentation of faces stopped after the first selection of a face^35,36^. However, the standard police procedure is to continue presenting faces even after the first identification to avoid the problematic case that the lineup stops before the eyewitness has seen the face of the suspect^29,37^. To enhance the external validity of the findings, the lineups used here continued even after participants had selected a face. If participants made more than one selection in a single lineup, later selections were considered revisions of earlier selections. If no face in the lineup was selected, the lineup was categorized as rejected.

## Results

The observed response frequencies that formed the basis of our analysis are reported in Table 1. To analyze these data, four instances of the model illustrated in Fig. 1 were needed: one for the simultaneous lineups with AI-generated filler images, one for simultaneous lineups with database-derived filler photographs, one for sequential lineups with AI-generated filler images and one for sequential lineups with database-derived filler photographs. Goodness-of-fit tests and parameter estimations were performed using multiTree^38^.

**Table 1:** Observed response frequencies for culprit identifications, filler selections and false lineup rejections in culprit-present lineups and innocent-suspect selections, filler selections and correct lineup rejections in culprit-absent lineups as a function of filler type (AI-generated images, database-derived photographs) in simultaneous and sequential lineups.

	Simultaneous lineups		Sequential lineups	
	AI-generated filler images	Database-derived filler photographs	AI-generated filler images	Database-derived filler photographs
Culprit-present lineups				
Culprit identifications	122	127	127	135
Filler selections	137	97	175	168
False lineup rejections	143	144	66	91
Culprit-absent lineups				
Innocent-suspect selections	22	36	37	54
Filler selections	170	129	225	201
Correct lineup rejections	210	203	106	139

**Figure 2:**
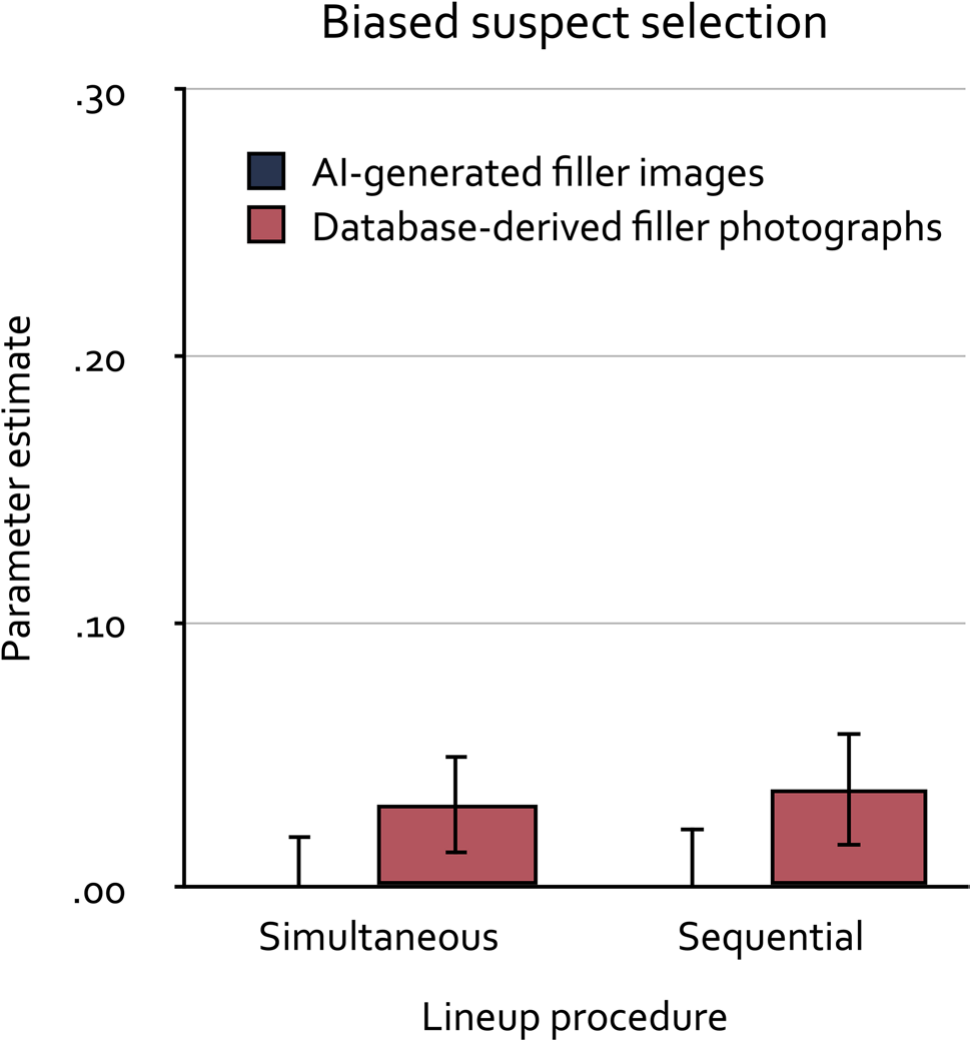
Estimates of parameter *b* representing the biased selection of the suspect as a function of filler type (AI-generated images, database-derived photographs) in simultaneous and sequential lineups. The error bars represent standard errors.

As in the validation experiment^13^ and the reanalyses^24^ targeting lineup fairness, parameter *dA* was set to be equal across conditions in the base model because culprit-absence detection was not expected to differ among conditions. Furthermore, given that all lineups contained six persons, the likelihood that, if guessing occurs, the random sampling results in the selection of the suspect was set to 0.16667 which simply is the reciprocal of the lineup size. The base model incorporating these restrictions fit the data, *G*^2^(3) = 5.90, *p* = 0.116. Parameter *dA* was estimated to be 0.07 (*SE* = 0.03). The estimates of parameter *b* are shown in Fig. 2. The estimates of parameters *dP* and *g* are reported in Table 2.

**Table 2:** Estimates of the parameters for culprit-presence detection (*dP*) and guessing-based selection (*g*) as a function of filler type (AI-generated images, database-derived photographs) in simultaneous and sequential lineups. Values in parentheses represent the standard errors.

	Simultaneous lineups		Sequential lineups	
	AI-generated filler images	Database-derived filler photographs	AI-generated filler images	Database-derived filler photographs
Culprit-presence detection (*dP*)	0.24 (0.03)	0.27 (0.03)	0.25 (0.03)	0.23 (0.03)
Guessing-based selection (*g*)	0.52 (0.02)	0.46 (0.02)	0.76 (0.02)	0.69 (0.02)

Multinomial processing tree models allow testing hypotheses directly at the level of the postulated processes^18^. The hypothesis that biased suspect selection does not differ between lineups with AI-generated filler images and lineups with database-derived filler photographs can be tested by setting parameter *b* to be equal across these conditions. If the model including this equality restriction provides a worse fit to the data than the base model, the difference test statistic ∆*G*^2^ increases. If this test statistic indicates a significant deviation between the base model and the nested restricted model, then it is necessary to conclude that parameter *b* differs between conditions^39^. The model with this equality restriction indeed had to be rejected because it provided a significantly worse fit to the data than the base model, ∆*G*^2^(2) = 6.42, *p* = 0.040, leading to the conclusion that the probability with which biased selection of the suspect occurs differed between the lineups with AI-generated filler images and the lineups with database-derived filler photographs. As is evident from Fig. 2, the lineups with AI-generated filler images were even fairer than the lineups with database-derived filler photographs, leading to even less biased selection of the suspect in the former than in the latter. The size of this effect is limited by the already low unfairness in the conditions with database-derived filler photographs, not by a lack of fairness in the conditions with AI-generated filler images. In fact, the biased-suspect-selection parameter *b* was indistinguishable from zero in the conditions with AI-generated filler images, ∆*G*^2^(2) < 0.01, bootstrapped *p* = 0.904, implying that the lineups with AI-generated filler images were perfectly fair. By contrast, at a descriptive level the biased-suspect-selection parameter *b* was slightly above zero in the conditions with database-derived filler photographs and this difference from zero was statistically significant, ∆*G*^2^(2) = 6.87, bootstrapped *p* = 0.008, implying that the lineups with database-derived filler photographs were nearly but not perfectly fair. For the latter two tests, we relied on the parametric bootstrap procedure implemented in multiTree^38^ to obtain an estimate of the *p* value from a simulated exact distribution because the null hypothesis implies a parameter value at the boundary of the parameter space [cf.^39,40^].

The manipulation of the filler type affected not only the biased-suspect-selection parameter *b* but also the guessing-based-selection parameter *g*, ∆*G*^2^(2) = 13.12, *p* = 0.001. The probability of guessing-based selection was higher in the lineups with AI-generated filler images than in the lineups with database-derived filler photographs. This is parallel to how manipulations of filler-to-suspect similarity affected the probability of guessing-based selection in our previous experiments on lineup fairness^13,14^ as well as in the reanalyses of previously published data^24^ in which a decrease of the probability of biased suspect selection was accompanied by an increase in the probability of guessing-based selection among all of the lineup members in fair compared to unfair lineups. In contrast, the probability of culprit-presence detection did not differ between the lineups with AI-generated filler images and the lineups with database-derived filler photographs, ∆*G*^2^(2) = 0.84, *p* = 0.658, leading to the conclusion that the use of the AI-generated filler images did not interfere with the detection process.

As a side note, the present design not only allows to compare lineups with AI-generated filler images and lineups with database-derived filler photographs but also to compare simultaneous and sequential lineups. The results reported in Table 2 show a pattern that is completely parallel to our previous experiments using the same paradigm^13,29^. Simultaneous and sequential lineups lead to equally good culprit-presence detection. Furthermore, simultaneous lineups lead to less guessing-based selection than sequential lineups. This is a typical finding for sequential lineups without “first yes counts” instructions in which comparatively high rates of guessing-based selections have been observed^29,37^. Given that these findings are secondary to the main research question, we will not elaborate on them here. Instead, we direct readers interested in these nuances to the comprehensive discussions of parallel findings in prior research^13,29^.

## Discussion

The most important finding of the present experiment is that, even with the current state of AI technology, it is already possible to generate facial filler images which lead to perfectly fair lineups. Using the 2-HT eyewitness identification model^13,24^, the probability of biased selection of the suspect in the lineups with AI-generated filler images was indistinguishable from zero, implying that biased suspect selection was completely absent because the lineups with AI-generated filler images were perfectly fair. This finding is all the more impressive considering that the biased-suspect-selection parameter of this measurement model has been demonstrated time and again to sensitively capture differences in the unfairness of lineups^13,24^ even if these differences are relatively subtle^14^. What is more, the probability of biased suspect selection was even lower in the lineups with AI-generated filler images than in the lineups with database-derived filler photographs that have been used in the fair control conditions in the previous experiments^13,14^. This difference between AI-generated filler images and database-derived filler photographs was small but considering that the probability of biased suspect selection was essentially zero in the conditions with AI-generated filler images, the difference is obviously limited by the already considerable fairness of the lineups with database-derived filler photographs, not by a lack of fairness of the lineups with AI-generated filler images.

As a side note, the beneficial effects of AI-generated filler images can also be observed at the level of the raw response rates. The proportion of innocent-suspect identifications was decreased in lineups with AI-generated filler images compared to lineups with database-derived filler photographs in both simultaneous (0.05 compared to 0.10) and sequential lineups (0.10 compared to 0.14). This is the consequence of the reduction of biased suspect selection in the lineups with AI-generated filler images in comparison to the lineups with database-derived filler photographs.

The lineups with AI-generated filler images did not only differ in the biased-suspect-selection parameter from the lineups with database-derived filler photographs but also in the parameter reflecting guessing-based selection among all of the lineup members. The obvious explanation of this data pattern is that a higher likeness of the fillers to the suspect in the lineups with AI-generated filler images provoked more guessing-based selection among all of the lineup members compared to the lineups with database-derived filler photographs [cf.^13,14,24^]. Per se a reduction in biased suspect selection combined with an increase in guessing-based selection among all of the members of the lineups with AI-generated filler images is a desirable pattern because it is the main benefit of fair lineups that non-memory-based selections are evenly distributed among all lineup members instead of being focused on the suspect [cf.^41–43^]. An additional benefit of this pattern is that, if desired, guessing-based selections can easily be reduced by appropriate pre-lineup instructions without affecting other processes such as culprit-presence detection^13^.

Given the rapid improvements in AI technology, the present findings are encouraging with regard to the potentials of AI image generation for constructing fair lineups. This is particularly true considering that AI image generation offers pragmatic advantages such as allowing police practitioners to generate filler images directly from the description of the culprit using text-to-image generation, avoiding the violation of identity rights of natural persons who are not suspects and eliminating the constraints of being bound to a database with a limited set of photographs. Now that the present results have established that the current state of AI technology is so advanced that lineups with AI-generated filler images can be perfectly fair and, in fact, better than lineups with database-derived filler photographs, future research should explore the boundary conditions for obtaining high-quality lineups with AI-generated filler images. The fact that AI image generation, in principle, allows to produce images of faces with any feature could be particularly useful when the suspect’s face has unusual features such as scars, tattoos and piercings or when the suspect belongs to a minority group so that it is unlikely that databases contain enough photographs of faces with the same features^12^. Here it could be an advantage that practitioners are no longer limited to a specific collection of faces when using AI image generation. However, it should not be taken for granted that AI-generated images of faces of high-enough quality can be obtained if the to-be-generated facial features are rare and therefore unlikely to be present in the AI training data with a high prevalence. Therefore, the quality of the output of the AI image generation must be rigorously monitored to responsibly implement the use of AI image generation as a tool for the creation of lineup fillers in practice.

In sum, the present experiment provides encouraging findings about the potential advantages of AI image generation for creating fair lineups. AI technology is rapidly progressing but, even at the present state of AI technology, the lineups with AI-generated filler images were perfectly fair and, in fact, better than lineups with database-derived filler photographs. It can be expected that AI image generation will become even more accessible and easier to use in the future. We hope that the present results stimulate more systematic research on how the vast potential of AI can be used to improve forensic practice.

## Data Availability

The data of the experiment and supplementary material are available at the project page of the Open Science Framework under https://osf.io/p5yz6/.
